# Evaluation of nine genotypes of oilseed rape (*Brassica napus* L.) for larval infestation and performance of rape stem weevil (*Ceutorhynchus napi* Gyll.)

**DOI:** 10.1371/journal.pone.0180807

**Published:** 2017-07-07

**Authors:** Heike L. Schaefer, Haiko Brandes, Bernd Ulber, Heiko C. Becker, Stefan Vidal

**Affiliations:** 1 Department for Crop Sciences, Division of Plant Pathology and Plant Protection, Section of Agricultural Entomology, Goettingen, Georg-August University, Germany; 2 Department of Crop Sciences, Section of Plant Breeding, Goettingen, Georg-August University, Germany; Chinese Academy of Agricultural Sciences Institute of Plant Protection, CHINA

## Abstract

The rape stem weevil, *Ceutorhynchus napi* Gyll., is a serious pest of winter oilseed rape (*Brassica napus* L.) crops in Europe causing severe yield loss. In currently used oilseed rape cultivars no resistance to *C*. *napi* has been identified. Resynthesized lines of *B*. *napus* have potential to broaden the genetic variability and may improve resistance to insect pests. In this study, the susceptibility to *C*. *napi* of three cultivars, one breeding line and five resynthesized lines of oilseed rape was compared in a semi-field plot experiment under multi-choice conditions. Plant acceptance for oviposition was estimated by counting the number of *C*. *napi* larvae in stems. The larval instar index and the dry body mass were assessed as indicators of larval performance. The extent of larval feeding within stems was determined by the stem injury coefficient. Morphological stem traits and stem contents of glucosinolates were assessed as potential mediators of resistance. The resynthesized line S30 had significantly fewer larvae than the cultivars Express617 and Visby and the resynthesized lines L122 and L16. The low level of larval infestation in S30 was associated with a low larval instar and stem injury index. Low numbers of larvae were not correlated with the length or diameter of stems, and the level of stem glucosinolates. As indicated by the low larval infestation and slow larval development the resistance of S30 to *C*. *napi* is based on both antixenotic and antibiotic properties of the genotypes. The resynthesized line S30 should therefore be introduced into *B*. *napus* breeding programs to enhance resistance against *C*. *napi*.

## Introduction

The rape stem weevil, *Ceutorhynchus napi* Gyll. (Coleoptera, Curculionidae), is a serious pest in European crops of winter oilseed rape (OSR, *Brassica napus* L.) [1]. Egg deposition by *C*. *napi* into elongating stems and larval feeding within the stem pith may cause significant yield losses [2]. OSR cultivars resistant to *C*. *napi* and other insect pests can comprise an important component of integrated pest management and are urgently needed to minimize the number of insecticide applications in Europe, where over-reliance on insecticide use has led to increasing incidences of insecticide resistance in OSR pests [3, 4]. Further, climate change might increase the risk of missing the proper insecticide application time, due to shifting the natural spring migration of *C*. *napi* towards an earlier onset of the invasion into the crop [5]. Interspecific interactions between insects with the same ecological niche can lead to the displacement of a species from a habitat by another. In those interactions insecticide applications can sometimes play an important role [6].

Brassicaceous genotypes have been screened for resistance traits against *C*. *napi* in only one study [7]. Several studies have emphasized the potential of resynthesized lines as sources of traits to enhance resistance to diseases [8, 9] and insect pests [7, 10]. Resynthesized lines have been developed by interspecific crossing of the two progenitor species of *B*. *napus*, *Brassica oleracea* L. and *Brassica rapa* L., and are used to broaden the genetic variability in OSR [11, 12]. For instance, resynthesized lines of *B*. *napus* are genetic sources for modifying profiles of glucosinolates [13], important plant defense compounds in Brassicaceae [7, 14, 15]. Therefore, the utilisation of resynthesized lines offers a promising opportunity to screen for sources of resistance to *C*. *napi* and other pests of OSR. Reduced performance and increased mortality (antibiosis) or reduced host location and acceptance (antixenosis) may result in plant resistance to pest insects [16, 17]. Further important means to control insect pests of OSR might be trap crops [18, 19] and/or the enhancement of natural enemies of these pests [20–22]. Also RNA interference is an important approach for managing coleopteran pests [23].

The life cycle of *C*. *napi* provides opportunities to assess potential resistance traits that act either on adult or on larval stages of the pest (or both). In early spring, adults of *C*. *napi* migrate from previous years fields of OSR to new OSR crops [24, 25]. In March-April, after approximately two weeks of feeding, females deposit single eggs into elongating stems. The three larval instars feed within the pith of stems for three to five weeks [24–26].

In stems of OSR, *C*. *napi* shares the same habitat and food resource with other stem-boring pests, such as cabbage stem weevil (*C*. *pallidactylus* (Marsh.)) and cabbage stem flea beetle (*Psylliodes chrysocephala* L.) [27, 28]. Many studies, investigating plant-herbivore interactions at multiple infestation situations, found that host plants damaged or pre-infested by one herbivore species were more or less attractive for another species [28–32]. To avoid inter-specific interactions between *C*. *napi* and other crucifer pests, in the present field study the tested OSR genotypes were therefore protected from natural infestation by insect proof cages.

Herbivores that specialise in crucifers make use of host plant volatiles to locate [33] and accept their hosts [34, 35]. Host location and acceptance of insect herbivores on brassicaceous crops can be affected by herbivory induced host plant volatile changes [33, 36] and altered glucosinolate contents [37, 38]. These volatiles are often the breakdown products of glucosinolates following cell disruption by herbivory [36].

Additionally, as obligate stem feeders the larvae of *C*. *napi* are also vulnerable to defence compounds in the host plant chosen by their mother. Chief among these are also the glucosinolates. In brassicaceous plants, glucosinolates may provide an effective defence against non-specific insect herbivores [15] but even *Brassica* specialists can be negatively affected by specific glucosinolates [14], e.g. *C*. *pallidactylus* [39], cabbage root fly (*Delia radicum* (L.)) [40] and cabbage flea beetles (*Phyllotreta* spp.) [41].

Beside secondary plant metabolites (e.g. glucosinolates) morphological plant traits can affect host plant acceptance and might be manipulated to achieve herbivore resistance in *B*. *napus* [17]. Females of *C*. *napi* preferably deposit their eggs into plant stems up to 22 cm long, whereas longer stems are frequently rejected [42]. Additionally, infestation by *C*. *napi* was reported to be significantly higher in sturdy compared to thin plant stems [43].

The objective of this study was to explore susceptibility of resynthesized lines of *B*. *napus* as sources of genetic resistance to *C*. *napi* in *B*. *napus* breeding. The infestation and performance of *C*. *napi* larvae were compared among nine *B*. *napus* genotypes, comprising three cultivars, one breeding line, and five resynthesized lines of *B*. *napus*. In order to avoid disturbing effects by multiple pest infestation of the tested genotypes on preference and performance of *C*. *napi*, e.g. changes of glucosinolate profiles, the experiment was conducted under protected semi-field conditions. The length and basal diameter of plant stems and glucosinolate profiles were measured.

## Materials and methods

### Plant genotypes in semi-field experiment

The semi-field experiment was conducted at the experimental station of Georg-August University, Goettingen, Germany (N51°33’53.8 E9°56’48.8) in 2011–2012. Nine genotypes, comprising five resynthesized lines, three cultivars and one breeding line of OSR, were selected based on their broad genetic background and variability of the glucosinolate content according [13, 44] (Table 1). Three additionally sown genotypes (R53, DH Samourai, Olimpiade) were severely affected by overwintering mortality and could not be included into further analyses. Single rows (length 2 m, 20 seeds m^-1^, 25 cm row spacing) of each genotype were sown on August 18^th^ 2011. In each of the six replicated plots, one row of each genotype was arranged at random. To reduce border effects, two rows of the winter OSR cultivar Krypton were sown at the margins of the plots. In order to avoid natural infestations of plants by other insect pests, each plot was enclosed by an insect-proof gauze cage (Seran PVDC, mesh width 425 μm) measuring 4.0 m × 2.0 m × 1.8 m, which was installed before natural crop colonisation by *C*. *napi* on February 22^nd^ 2012.

**Table 1:** Genotypes of *Brassica napus* selected for evaluation of susceptibility to *Ceutorhynchus napi* in 2011–2012.

Genotype / cultivar	Type	Species
**Campala**	Winter rape, cultivar	*B*. *napus* var. *biennis*
**Goe1991**	Line	*B*. *napus* var. *biennis*
**Express617**	Winter rape, cultivar	*B*. *napus* var. *biennis*
**Visby**	Winter rape, hybrid cultivar	*B*. *napus* var. *biennis*
**G53**	Resynthesized line	*B*. *oleracea* convar. capitata var. *capitata* X *B*. *rapa* ssp. nipposinica var. *perviridis*
**S3**	Resynthesized line	*B*. *rapa* ssp. rapa X *B*. *oleracea* convar. acephala var. *sabellica*
**L122**	Resynthesized line	*B*. *oleracea* convar. capitata var. *sabauda* X *B*. *rapa* ssp. pekinensis
**S30**	Resynthesized line	*B*. *olerace*a convar. capitata var. *capitata* X *B*. *rapa* ssp. pekinensis
**L16**	Resynthesized line	*B*. *oleracea* convar. botrytis var. *alboglabra* X *B*. *rapa* ssp. pekinensis

### Larval infestation and performance

Adult rape stem weevils used for targeted release into cages were collected from non-sprayed previous year’s OSR fields on March 3^rd^, and maintained in plastic boxes in a climatic chamber at 6°C (L16:D8). Adults were supplied with leaves of the OSR cultivar Mozart grown in a glasshouse. In order to adjust the start of the experiment, the natural spring migration of *C*. *napi* was monitored with yellow traps containing water and some droplets of a detergent. The traps were installed in OSR crops adjacent to the semi-field experiment. On March 23^rd^, at the beginning of natural spring migration of *C*. *napi* into the new OSR crops and simultaneously with stem elongation of the genotypes, 60 females and 30 males of post-diapause *C*. *napi* adults were released into each cage.

On May 8^th^, at full-flowering (BBCH growth stage 64–67), ten randomly selected plants were collected from each plot and genotype. The main stems were dissected under a stereo microscope (Zeiss, Stemi 2000-C) to count the number of *C*. *napi* larvae. The number of larvae was assessed only in the main stems, because the high number of plant samples had to be analysed before the plants started wilting or moulding. Therefore the number of larvae in side shoots could not be assessed in this study. The larval dry body mass and the larval instar index were evaluated as indicators of the rate of larval development in the different plant genotypes. The dry body mass of ten randomly selected larvae (killed with 70% EtOH) per larval instar, genotype and plot was assessed by drying at 60°C for three days and weighing individual larvae (Sartorius micro scale, MC5). Discrimination between the three larval instars of *C*. *napi* was based on the head capsule width [25]. The progress of the larval development in the stems of different plant genotypes was assessed by the larval instar index, which was calculated by subtracting the number of 2^nd^ instar larvae from the number of 3^rd^ instar larvae and adding a constant (K = 2), to avoid negative values [45]. The value of the index increases as the proportion of 3^rd^ larval instars increases. To estimate the relative extend of larval feeding in the stems of tested plant genotypes the stem injury coefficient was calculated from plants sampled on May 8^th^, by subtracting the length of the larval feeding tunnel from length of the full-grown stem [46].

### Morphological traits of plants

On March 23^rd^, just before release of adult stem weevils into the cages, plant density of the genotypes was assessed by counting all plants per plot. On March 31^st^, five randomly selected plants were collected from each plot to assess the BBCH growth stage [47] and the length of main stems above ground. The interaction between the length and basal diameter of stems, the number of larvae per stem and the larval performance were determined from plants sampled on May 8^th^.

### Analysis of glucosinolate profiles of plants

In order to determine the effect of the glucosinolate content on the infestation of plant genotypes by *C*. *napi* five randomly selected non-infested main stems per genotype were collected per plot just before release of adult weevils on March 23^rd^ for glucosinolate analyses. The glucosinolate content was analysed on six of nine genotypes (Campala, Express617, Visby, L16, S3, S30) because plant material of three genotypes was not sufficient for the analyses. The stems were immediately frozen on dry ice and stored at -20°C. After freeze-drying for 96 hours, the stems were homogenised using a mill (Krups KM 75). Stem glucosinolates were separated and individual compounds were identified and quantified [48] using a Shimadzu Prominence LC20AT series HPLC (Shimadzu Deutschland GmbH) equipped with a Nucleodur 100–3 C18 column (Macherey Nagel‎). Desulfoglucosinolates were extracted as detailed in [13] and were separated using a water-acetonitrile gradient (solvent A water, solvent B acetonitrile; 0–20 min 1–20% B; 20–25 min 20% B; 25–27 min 20% B; 27–34 min 1% B) at a flow rate of 0.6 ml / min. Retention times of known standards were used to identify desulfoglucosinolates. The concentration of glucosinolates is expressed in μmol / g dry weight (DW).

### Data analysis

Univariate data analysis was done using Statistica 10 (StatSoft^®^, Tulsa, USA) and tested for normal distribution with the Shapiro-Wilk *W* test. Multivariate data analyses were done with R 3.0.1.

#### Relationships between genotype and *C*. *napi* infestation and performance

To remove the effect of plant stem length and basal diameter of stems of genotypes on the number of *C*. *napi* larvae present in stems on May 8^th^, analysis of co-variance (ANCOVA) was conducted. The number of larvae was treated as main factor and the length and the basal diameter of full-flowering stems were included into the model as co-variates. The plant density of genotypes and length of stems at the beginning of the infestation period were not included into the model, because of multi-collinearity with the covariate length of full-grown stems. The genotype effect on the stem injury coefficient was analysed by factorial one-way analysis of variance (ANOVA); the differences among means were evaluated by the Tukey-test. The effects of plant genotype on the length of larval feeding tunnels, the dry body mass of 2^nd^ & 3^rd^ larval instars, and on the larval instar index, respectively, were analysed by Kruskal-Wallis-test (KW-test).

A linear regression was used to test the effect of plant density, stem length (stem samples of May 8^th^) and basal diameter of stem on the number of larvae. The relationship between the length of stem of genotypes on March 31^st^ and number of larvae was not analysed because of multi-collinearity of length of stems of samples of March 31^st^ and May 8^th^ with the number of *C*. *napi* larvae. Pearson Product-Moment Correlation was used to test the relationship between the number of larvae and the larval instar index, the number of larvae and the stem injury coefficient and between length of stems sampled on March 31^st^ and May 8^th^. The correlation between the larval instar index and the stem injury coefficient was not analysed because of multi-collinearity of both, larval instar index and stem injury coefficient with the number of *C*. *napi* larvae. Linear regression was used to analyse the association between the basal diameters of full-flowering stems on the larval instar index.

To investigate the influence of non-infested stem glucosinolate profiles on the number of *C*. *napi* larvae in stems, partial least squares regression (PLSR) [49] was used. Data were scaled to unit variance, and were mean centred by default in the analyses. Again, the regression between the glucosinolate profiles and the stem injury coefficient was not analysed because of multi-collinearity of the stem injury coefficient with the number of larvae. For the same reason no regression was performed on data of the larval instar index.

#### Effect of genotype on plant traits

**Fig 1:**
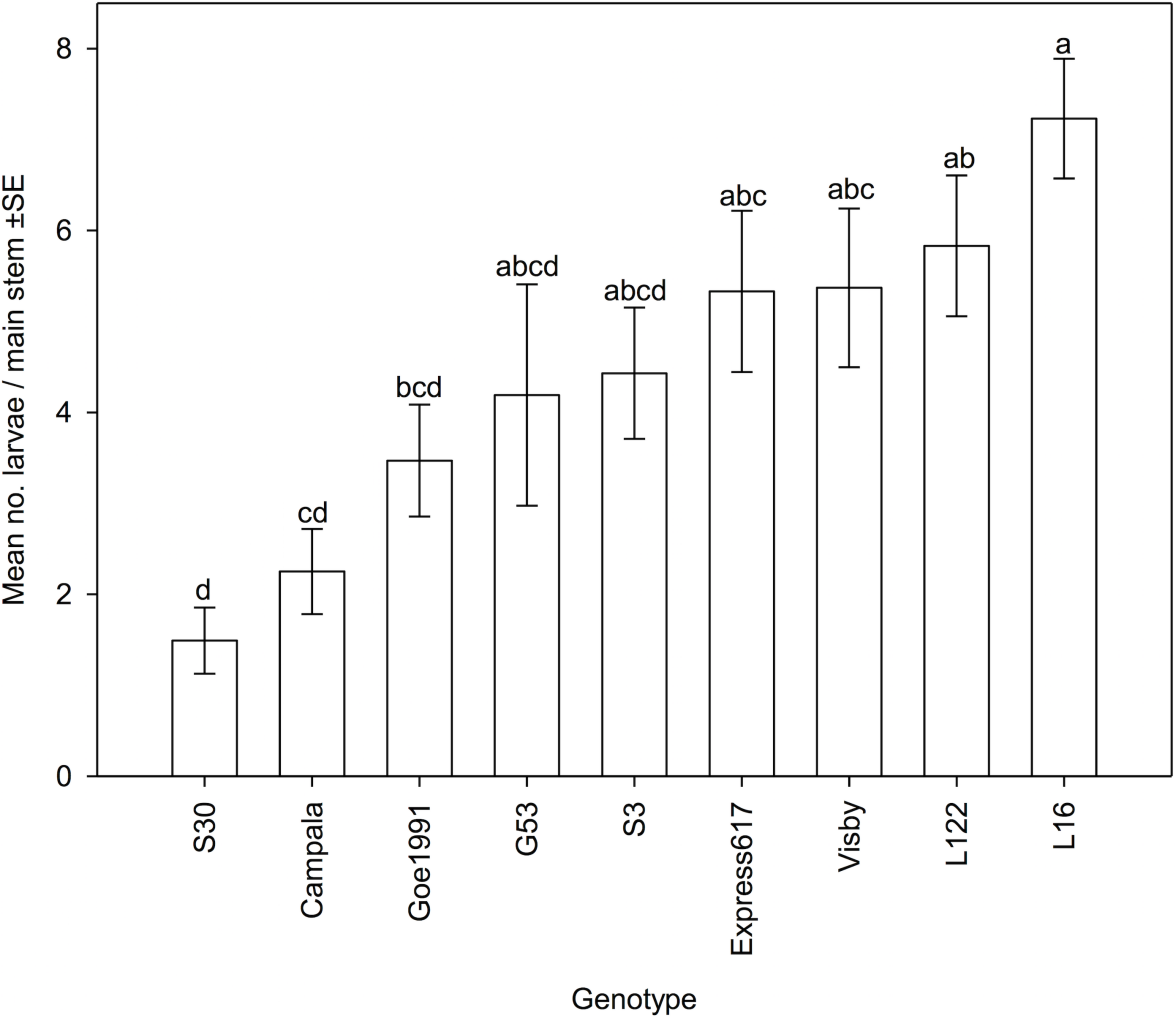
Number of *Ceutorhynchus napi* larvae counted in main stems of nine *Brassica napus* genotypes / cultivars. Mean values of six replicates, error bars are ±SE. Analysis of covariance (ANCOVA), *F*_8, 43_ = 7.148, *P* = 0.000; genotypes not followed by the same letter significantly differ, *P* ≤ 0.05, ANCOVA followed by Tukey-test.

The effects of plant genotype on plant density, length of full-flowering stems and on basal diameter of full-flowering stems (stem samples of May 8^th^) were analysed by ANOVA; the differences among means were evaluated by the Tukey-test. Lengths of full-flowering stems were log x+1 transformed to normalise the residuals. The length of stems at the beginning of the infestation period (stem samples of March 31^st^) was analysed differently from plant density, length of full-flowering stems and basal diameters because of non-normal distribution of residuals. The genotype effect on the length of stems at the beginning of the infestation period was analysed by KW-test. To test non-infested stems for between-genotype differences in glucosinolate profiles, partial least squares—discriminant analysis (PLS-DA) [50] was used, and significance of discrimination was tested by multivariate analysis of variance (MANOVA). To investigate differences among the content of individual glucosinolates of genotypes, glucosinolate profiles of all genotypes (responsible variables) were compared with a reference glucosinolate profile (reference). Glucosinolate profiles of genotypes S30, Campala and L16 were chosen in the analyses as reference as S30 and Campala being notable for a very low larval infestation and L16 for the highest *C*. *napi* larvae infestation (Fig 1).

## Results

### Responses of *C*. *napi* to plant genotypes

#### Number and performance of *C*. *napi* larvae

The number of *C*. *napi* larvae per stem was lowest in the resynthesized line S30 (Fig 1). Significant differences between genotypes were found (Table 2 and Fig 1), with S30 having significantly fewer larvae than the resynthesized lines L16 and L122 and the cultivars Visby and Express617 (Fig 1).

**Table 2:** Effect of genotype and covariates on the number of *Ceutorhynchus napi* larvae. Analysis of covariance, *P* ≤ 0.05. Nine genotypes, six replicates, sample size (*N*), degree of freedom (*DF*), and *F*-value.

	*N*	*DF*	*F*	*P*
Factor: Genotype / cultivar	54	8	7.148	0.000
Covariate: Basal diameter of full-grown stem	54	1	12.382	0.001
Covariate: Length of full-grown stem	54	1	8.377	0.006

**Fig 2:**
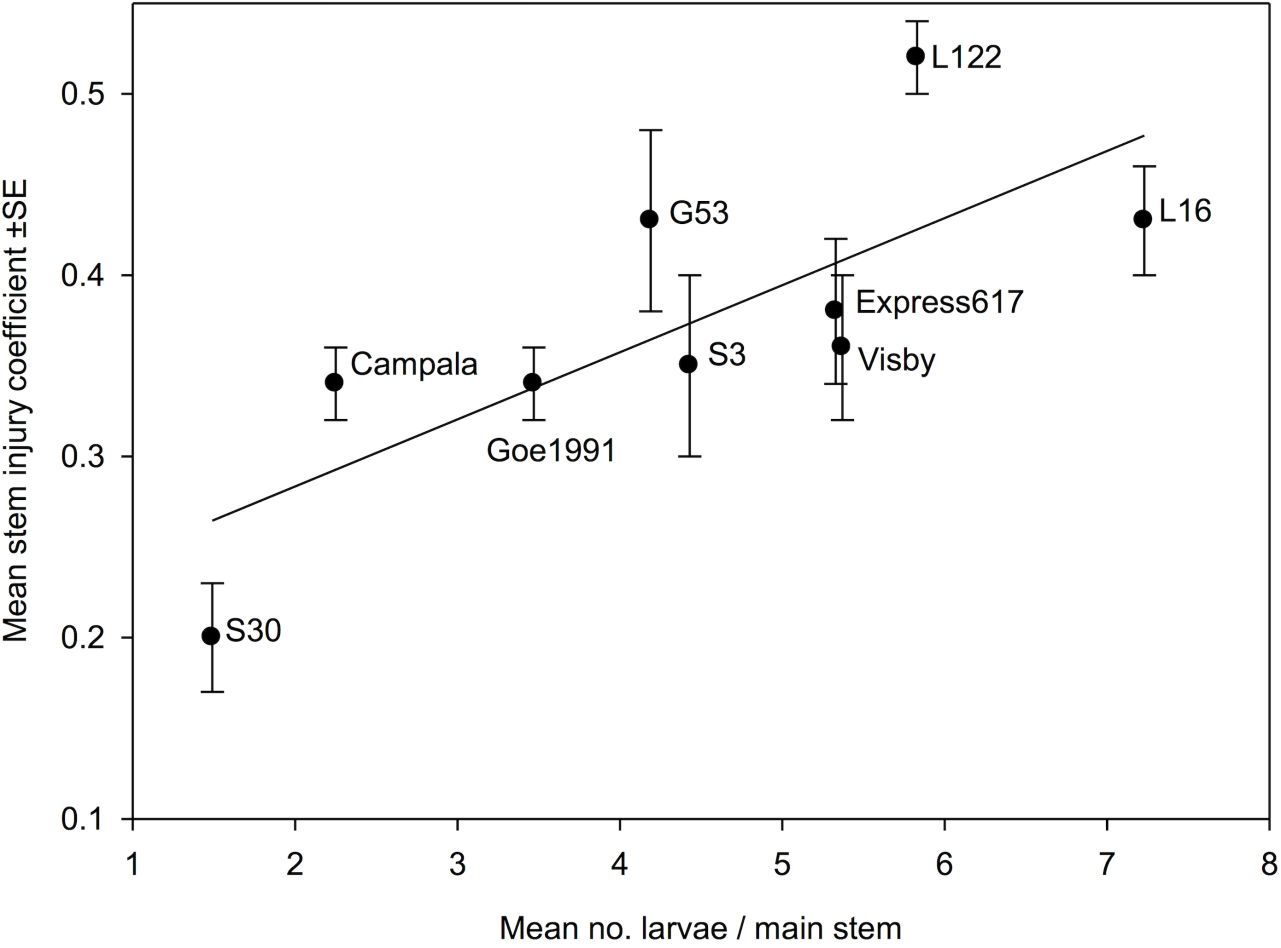
Correlation between the stem injury coefficients and the number of *Ceutorhynchus napi* larvae found in main stems of nine *Brassica napus* genotypes / cultivars. Pearson Product Moment Correlation: *R* = 0.763; *N* = 54, *P* = 0.017.

**Fig 3:**
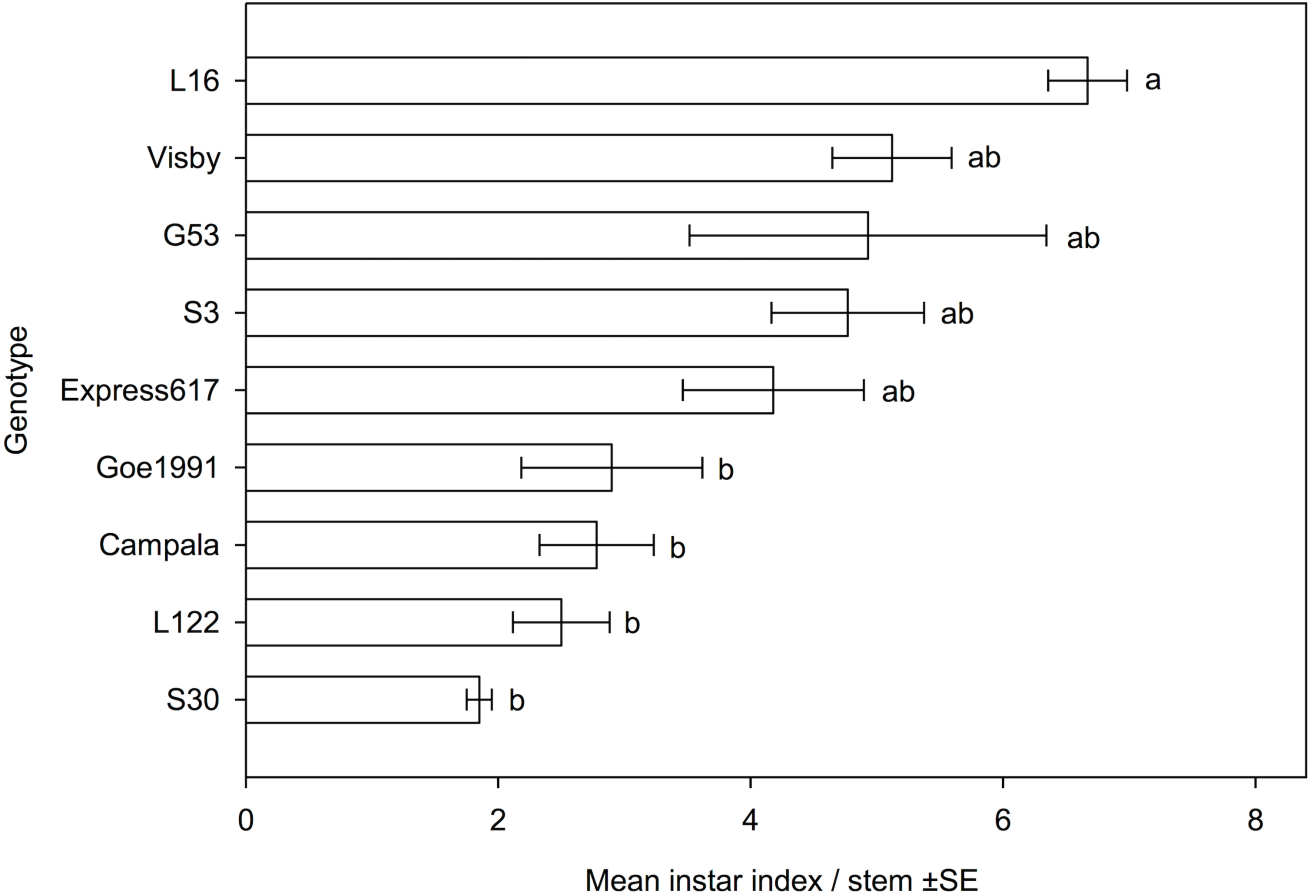
Comparisons of the larval instar indices for *Ceutorhynchus napi* larvae in the main stems of nine *Brassica napus* genotypes / cultivars. Mean values of six replicates, error bars are ± SE. Lower index values reflect a delayed larval development rate. Kruskal-Wallis test, *H* (8, *N* = 54) = 29.861, *P* = 0.000; genotypes not followed by the same letter significantly differ, *P* ≤ 0.05. Larval instar index = (L3—L2) + K; K = 2.

In accordance with the low number of larvae in S30, the length of the feeding tunnels per stem caused by *C*. *napi* larvae were very short in this line. S30 having significantly shorter feeding tunnels than L16 and Visby (Table 3). The relative amount of pith tissue consumed by *C*. *napi* larvae, as reflected by the stem injury coefficient, significantly differed between the genotypes (Table 3). The stem injury coefficient was lowest in the resynthesized line S30, the genotype with the smallest number of larvae, and was significantly lower compared to the resynthesized lines L122, L16 and G53, and cultivar Express617, respectively, the plant genotypes with a large number of larvae (Fig 1). The mean number of larvae per stem was significantly positively correlated with the stem injury coefficient (Fig 2). The accumulation of biomass of the 2^nd^ and 3^rd^ larval instar of *C*. *napi* was similar in each genotype, and the dry biomass of both 2^nd^ and 3^rd^ instar larvae did not differ significantly (Table 3). The rate of *C*. *napi* larval development, as reflected by the larval instar index, significantly differed between the genotypes (Fig 3). Larval development was most retarded in the resynthesized lines S30 and L122, cultivar Campala and breeding line Goe1991, with larval instar indices significantly smaller than in resynthesized line L16 in which larval development was fastest (Fig 3). The resynthesized lines S30 and L16, respectively, had the smallest and the largest number of larvae per stem (Fig 1). Over all genotypes, the number of larvae was significantly positively correlated with the larval instar index (*R* = 0.731, *N* = 54, *P* = 0.025).

**Table 3:** Length of feeding tunnels and stem injury coefficient of nine *Brassica napus* genotypes / cultivars infested by *Ceutorhynchus napi* larvae. Dry body mass of 2^nd^ and 3^rd^ larval instars developed in stems of these genotypes. Mean values (±SE).

Genotype / cultivar	Length of feeding tunnels (cm)	Stem injury coefficient	Dry body mass of 2^nd^ larval instar (mg)	Dry body mass of 3^rd^ larval instar (mg)
Campala	16.82 ± 2.12 ab	0.34 ± 0.02 bc	0.19 ± 0.02	1.19 ± 0.24
Goe1991	22.25 ± 3.35 ab	0.34 ± 0.02 bc	0.20 ± 0.03	0.83 ± 0.20
Express617	29.25 ± 2.10 ab	0.38 ± 0.04 ab	0.19 ± 0.04	1.28 ± 0.34
Visby	34.73 ± 4.10 a	0.36 ± 0.04 bc	0.33 ± 0.08	1.53 ± 0.26
G53	26.69 ± 5.25 ab	0.43 ± 0.10 ab	0.17 ± 0.01	1.52 ± 0.50
S3	29.73 ± 2.68 ab	0.35 ± 0.05 bc	0.20 ± 0.01	1.79 ± 0.33
L122	27.21 ± 2.78 ab	0.52 ± 0.02 a	0.24 ± 0.03	1.11 ± 0.19
S30	13.09 ± 1.77 b	0.20 ± 0.03 c	0.21 ± 0.02	1.31 ± 0.45
L16	34.45 ± 2.55 a	0.43 ± 0.03 ab	0.32 ± 0.11	1.87 ± 0.35
**Analysis of variance**				
ANOVA (*F*_8, 45_)	-	6.257	-	-
KW-test (*H* (8, *N* = 54))	27.058	-	8.361	10.243
	*P* = 0.001	*P* = 0.000	*P* = 0.399	*P* = 0.248

Within each column, genotypes not followed by the same letter significantly differ, *P* ≤ 0.05 (Length of feeding tunnels, Kruskal-Wallis test; stem injury coefficient, ANOVA followed by Tukey-test).

### Phenotypic differences between plant genotypes

#### Physical plant traits

On March 23^rd^, when *C*. *napi* adults were released into the cages, plant density significantly differed between the tested genotypes. The resynthesized line L122 showed significantly lower numbers of plants / m then all other plant genotypes (Table 4). Though plant density differed between genotypes, the mean number of *C*. *napi* larvae in stems was not significantly dependent upon plant density (*R* = 0.371, *F* = 1.116, *P* = 0.326).

**Table 4:** Plant density, BBCH growth stage, stem lengths and basal diameter of stems of nine *Brassica napus* genotypes / cultivars encompassing the infestation period of *Ceutorhynchus napi*. Mean values ± SE.

Genotype / cultivar	BBCH March 31^st^	Plant density (plants / m) March 23^rd^	Length of stem (cm) March 31^st^	Length of stem (cm) May 8^th^	Basal diameter of stem (cm) May 8^th^
Campala	52	6.08 ± 0.40 ab	17.08 ± 1.98 ab	48.82 ± 4.96 d	1.12 ± 0.10 abc
Goe1991	52	6.17 ± 0.67 b	16.88 ± 0.88 ab	63.58 ± 7.43 bcd	0.89 ± 0.10 bc
Express617	55	8.25 ± 0.38 ab	19.58 ± 2.77 ab	77.88 ± 6.04 abc	1.25 ± 0.09 ab
Visby	57	9.75 ± 0.53 a	37.92 ± 1.50 a	97.60 ± 3.48 a	1.39 ± 0.09 a
G53	52	6.25 ± 0.57 b	15.00 ± 3.26 b	58.74 ± 5.73 cd	0.77 ± 0.11 bc
S3	57	9.50 ± 0.66 a	30.42 ± 3.95 ab	89.80 ± 7.06 ab	0.96 ± 0.03 bc
L122	52	3.33 ± 0.89 c	13.50 ± 3.12 b	52.38 ± 4.85 d	1.05 ± 0.09 abc
S30	55	6.25 ± 0.42 b	23.74 ± 3.69 ab	63.60 ± 3.64 bcd	0.95 ± 0.09 bc
L16	52	9.83 ± 0.76 a	16.67 ± 1.79 b	81.55 ± 6.14 abc	1.07 ± 0.07 abc
**Analysis of variance**					
ANOVA (*F*_8, 45_)		13.087	-	8.857	4.567
KW-test *H* ((8, *N* = 54))		-	26.364		
		*P* = 0.000	*P* = 0.001	*P* = 0.000	*P* = 0.000

Within each column, genotypes not followed by the same letter significantly differ, *P* ≤ 0.05 (Stem length March 31^st^, Kruskal-Wallis test; Plant density, stem length May 8^th^ and basal stem diameter, ANOVA followed by Tukey-test).

There was little variation between the BBCH growth stages of plant genotypes on March 31^st^, at the beginning of the oviposition period of *C*. *napi* (Table 4). All plants were at early bud stage, with the resynthesized line S30 and cultivar Express617 (BBCH growth stage 55) and resynthesized line S3 and cultivar Visby (BBCH growth stage 57) slightly more advanced than the other genotypes (BBCH growth stage 52) (Table 4). Plant samples of March 31^st^ and May 8^th^ were chosen for analysis of differences between physical traits as these dates encompassed the infestation period of *C*. *napi* females. On each of these dates there was considerable variability in stem length and there were significant differences between the genotypes (Table 4). On March 31^st^, at the beginning of the infestation period, the resynthesized lines L122, G53 and L16 being notable for shortness and the cultivar Visby, at a slightly more advanced growth stage, having the longest stem (Table 4). Towards the end of the larval development of *C*. *napi*, when plant genotypes were full-flowering (stem samples of May 8^th^), the cultivar Visby being also notable for length and the cultivar Campala, the resynthesized lines L122, G53 and S30 and the breeding line Goe1991 having the shortest stem (Table 4). The ANCOVA revealed a significant interaction between the length of full-flowering stem (stem samples of May 8^th^) and the number of larvae in stems of genotypes (Table 2). However, the mean number of *C*. *napi* larvae in stems of May 8^th^ was not significantly dependent upon length of stem samples of May 8^th^ (*R* = 0.456, *F* = 0.021, *P* = 0.885). Additionally, differences in the length of stems at the beginning of the infestation of *C*. *napi* (stem samples of Mach 31^st^) had no influence on the number of larvae in stems of May 8^th^, since the length of stems of genotypes on March 31^st^ correlated significantly positively with the length of stems of samples on May 8^th^ (*R* = 0.828, *N* = 54, *P* = 0.000).

On May 8^th^, the basal diameters of stems were more variable within the genotypes. The cultivar Visby had a significantly more vigorous basal stem diameter than Goe1991, G53, S3 and S30 (Table 4). However, the mean number of *C*. *napi* larvae in stems of May 8^th^ was not significantly dependent upon basal stem diameter (*R* = 0.339, *F* = 0.911, *P* = 0.372). The mean larval instar index per genotype did also not significantly depend upon basal stem diameter (*R* = 0.190, *F* = 0.263, *P* = 0.624).

#### Glucosinolate profiles and their effects on *C*. *napi*

**Fig 4:**
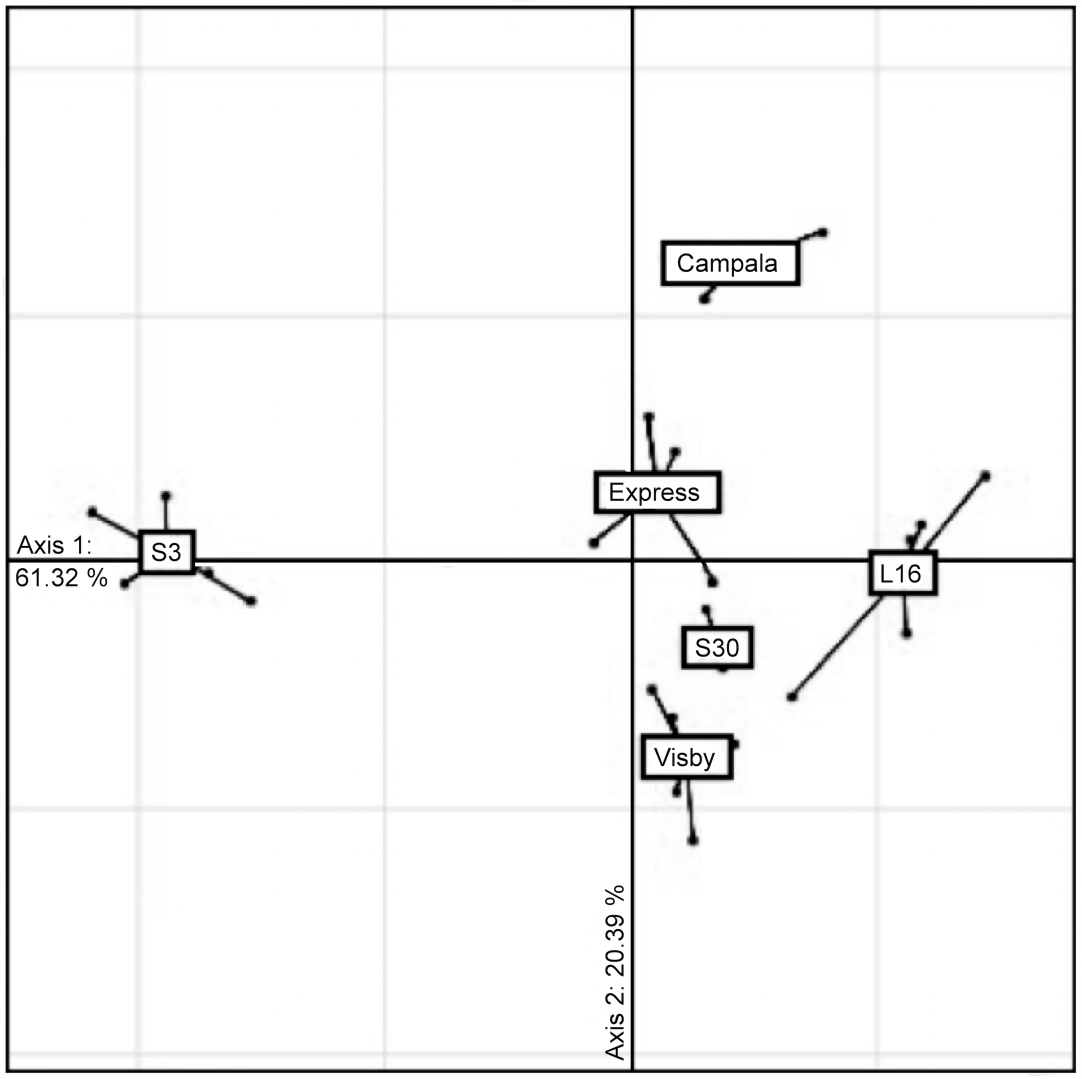
Partial least squares-discriminant (PLS-DA) analysis score plot for the glucosinolate profile of non-infested stems of six *Brassica napus* genotypes / cultivars sampled on April 17^th^, 81.71% of the intergenotypic variance explained. MANOVA test for the discrimination of genotypes: pseudo-*F*_45, 80_ = 3.124, *P* ≤ 0.05.

The glucosinolate content in non-infested stems showed a high variability between plant genotypes, with the genotype exhibiting the highest total glucosinolate content, S3, having more than five times as much as the lowest, L16 (Table 5). Glucosinolate profiles were also highly variable (Table 5) and the PLS-DA analysis showed that the profiles significantly differed between genotypes; the score plot explained 81.71% of intergenotypic variance. The cluster pattern in the PLS-DA score plot indicated that the glucosinolate profile of S3 was clearly distinct, falling outside a broad cluster containing the five other genotypes (Fig 4). By comparing the glucosinolate profiles of all genotypes with a reference glucosinolate profile, significant differences between the content of individual glucosinolates of genotypes were found. Glucosinolate profiles of S30, Campala and L16 were chosen in the analysis as references as S30 and Campala showed a very low larval infestation and L16 the highest larval infestation by *C*. *napi* (Fig 1).

**Table 5:** Glucosinolate contents of non-infested stems (μmol / g DW) on March 23^rd^, just before releasing *Ceutorhynchus napi* adults, of six *Brassica napus* genotypes / cultivars grown in the semi-field experiment in 2011–2012. Mean values of four to six replicates.

Genotype / cultivar	PRO	GNL	ALY	GNA	GBN	4OH	GBC	4ME	NEO	Total
Campala	6.71	0.00	1.38	1.67	9.51	0.02	3.25	0.26	4.05	26.85
Express617	7.56	0.03	1.68	1.10	7.05	0.01	1.57	0.24	2.52	21.76
Visby	5.45	0.00	1.89	0.52	3.23	0.00	0.98	0.10	0.61	12.78
S3	25.16	0.35	0.94	10.39	8.20	0.01	0.78	0.31	0.36	46.50
S30	6.79	0.00	1.33	0.49	4.40	0.01	1.73	0.09	1.32	16.16
L16	1.33	0.00	0.39	0.29	0.78	0.06	2.82	0.08	2.48	8.23

Aliphatic glucosinolates: PRO = progoitrin = 2-hydroxy-3-butenyl, GNL = gluconapoleiferin = 2-hydroxy-4-pentenyl, ALY = glucoalyssin = 5-methylsulphinylpentyl, GNA = gluconapin = 3-butenyl, GBN = glucobrassicanapin = 4-pentenyl; Indolyl glucosinolates: 4OH = 4-hydroxyglucobrassicin = 4-hydroxy-3-indolylmethyl, GBC = glucobrassicin = 3-indolylmethyl, 4ME = 4-methoxyglucobrassicin = 4-methoxy-3-indolylmethyl, NEO = neoglucobrassicin = 1-methoxy-3-indolylmethyl.

The PRO glucosinolate content of non-infested stems of S30 significantly differed from L16 and S3. The content of GBN in S30 significantly differed from L16, Express617, Campala, and S3. The content of GBC in S30 significantly differed from Campala. The content of NEO in S30 significantly differed from Camapla, Express617, L16 and S3 (Table 5 and Fig 4).

The PRO glucosinolate content of non-infested stems of Campala differed significantly from L16 and S3. The content of GNA in Campala significantly differed from S30 and also from Visby, S3 and L16. The content of GBN in Campala significantly differed from S30 and also from Visby, Express617 and L16. The content of GBC in Campala significantly differed from S30 and also from Visby, Express617 and S3. The content of NEO in Campala significantly differed from S30 and also from Visby, Express617, S3 and L16 (Table 5 and Fig 4).

The 4OH glucosinolate content of non-infested stems of L16 significantly differed from S30 and Campala, but also from Visby, Express617 and S3. The content of GBC in L16 significantly differed from Visby, Express617 and S3. The content of NEO in L16 significantly differed from S30 and Campala and also from Visby and S3 (Table 5 and Fig 4).

**Fig 5:**
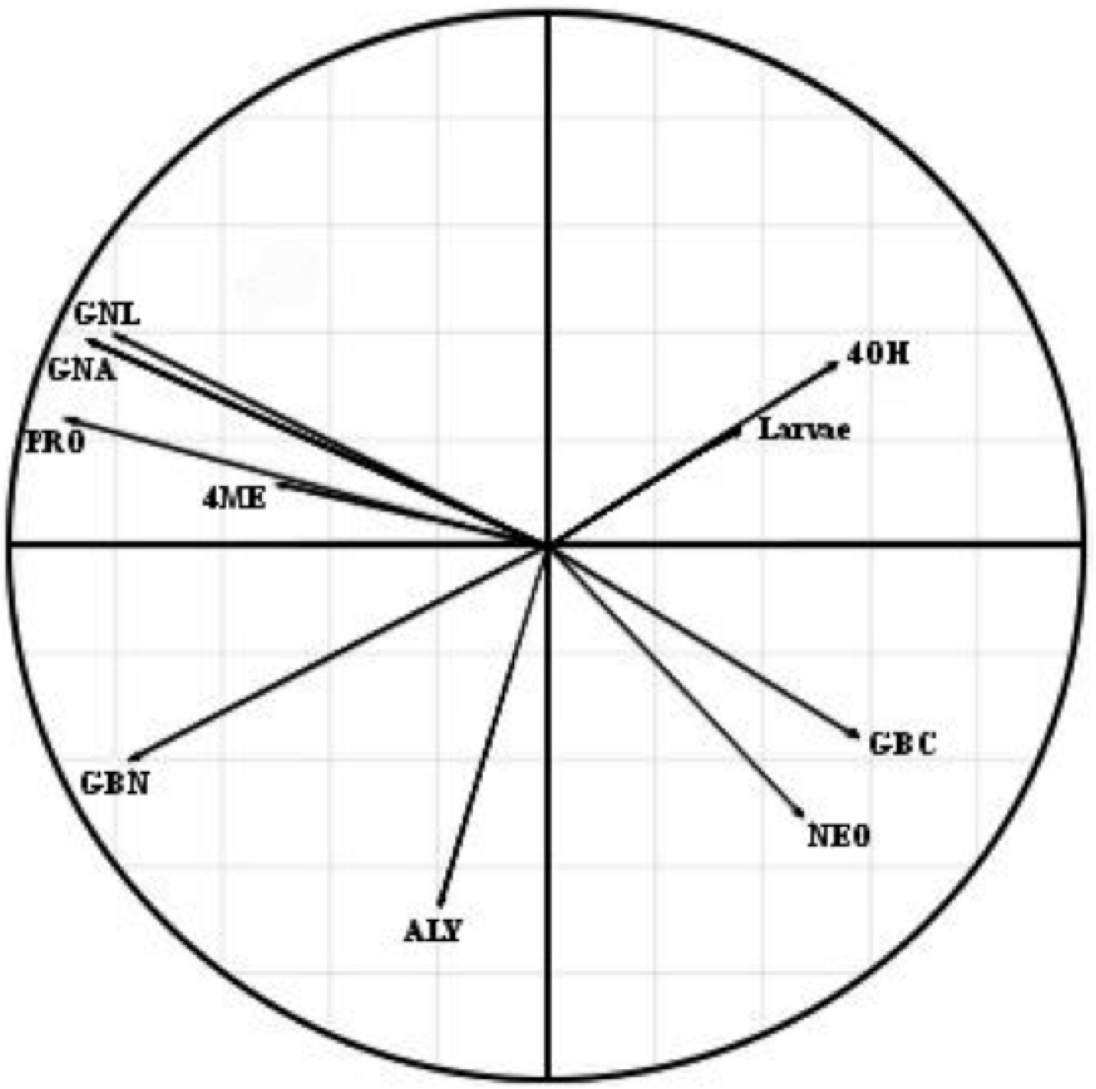
Partial least squares-regression (PLSR) analysis loading plot showing relationships between the glucosinolate profile of non-infested stems of six *Brassica napus* genotypes / cultivars on March 23^rd^ and the number of *Ceutorhynchus napi* larvae counted per main stems on May 8^th^. The factorial map 1–2 explained 18.11% of larval count variance. Low variances (≤ 50%) are unrepresentative in the PLSR analysis. See legend of Table 5 for key to abbreviations for glucosinolates.

The content of both 4OH and NEO in L16 differed from those of S30 and Campala. Due to this great variation between the glucosinolate profiles of genotypes and between levels of individual glucosinolates in non-infested stems, PLSR was chosen for analysis to investigate the influence of stem glucosinolate profiles on the number of *C*. *napi* larvae. The PLSR analysis (Fig 5) revealed a low larval count variance (≤ 50%) and a short larval arrow (≤ radius), thereby indicating no close relationship between the glucosinolate profile of non-infested stems and the number of larvae in stem samples (Fig 5). The arrows of the factorial map (≤ radius) were not found to be representative in the PLSR analysis.

## Discussion

This semi-field study demonstrated the potential of resynthesized lines of *B*. *napus* as potential sources of resistance against the winter OSR pest *C*. *napi*. We found a remarkable variation in larval infestations in the plant material tested and identified the resynthesized line S30 to be one of the very low infested genotypes, comprising five resynthesized lines, one breeding line and three commercial cultivars of OSR. S30 showed resistance against *C*. *napi* not only under protected semi-field conditions when effects by multiple pest infestation have been avoided, but also under field conditions when potential resistance-related plant traits were affected by various other pest species [7].

### Responses of *C*. *napi* to plant genotypes

#### Number and performance of *C*. *napi* larvae

Despite considerable variation in the number of *C*. *napi* larvae found in main stems both within and between genotypes, plant infestation by larvae was very low in the resynthesized line S30 (Fig 1). As described in detail previously [7], S30 is likely to be a useful resource of resistance traits for breeding of *B*. *napus*. Additionally, Eickermann and Ulber [10] found a low level of infestation by *C*. *pallidactylus*, another stem-boring pest of OSR, in the line S30. The low number of larvae in S30 may have resulted from decreased oviposition by *C*. *napi* females or from increased egg and / or larval mortality. Low numbers of *C*. *napi* eggs deposited in stems of S30 would indicate an antixenosis resistance mechanism, while a high egg and / or larval mortality would indicate antibiosis resistance. The main period of oviposition by *C*. *napi* encompasses approximately three weeks [7]. Therefore, a reliable assessment of the number of eggs of genotypes would have required a repeated sampling design of the plant stems in this semi-field experiment. However, plant density was too low to allow several samplings. Due to this constraint, counts of *C*. *napi* larvae were used to estimate host plant acceptance of the genotypes. Another open field experiment comparing the number of eggs deposited by *C*. *napi* into nine genotypes of *B*. *napus* throughout the oviposition period provided evidence that females laid significantly fewer eggs into S30 [7].

The stem injury coefficient, reflecting the extent of larval feeding within stems varied considerably between the tested genotypes and was smallest in the resynthesized line S30, the line least infested by *C*. *napi* larvae (Table 3). However, the stem injury coefficient of S30 did not statistically differ from Campala, Goe1991, Visby and S3. The number of larvae per stem was positively correlated with the stem injury coefficient (Fig 2). Therefore, the stem injury coefficient can provide a useful parameter for rapid screening of a large assortment of *B*. *napus* genotypes / cultivars for infestation by *C*. *napi* larvae. Eickermann et al. [39] found that the number of the larvae of *C*. *pallidactylus* is positively correlated with the stem injury coefficient. The stem injury coefficient caused by the larvae of *P*. *chrysocephala* was associated with yield loss of OSR [46].

The dry body mass of larval instars did not significantly differ between genotypes (Table 3). This indicates that different concentrations of primary metabolites, such as proteins and carbohydrates, in stem tissue are unlikely to have an effect on infestation by *C*. *napi* larvae in this study. Comparisons of larval instars within stems suggested that line S30 might also exhibit antibiosis resistance against *C*. *napi* larvae. As described in detail previously [7], this line had the lowest value of the instar index among all genotypes tested, with only a small proportion of larvae having reached L3 by May 8^th^ (Fig 3). Antibiosis is known to affect herbivore performance and can result in prolonged development [17]. However, a low larval instar index might also have resulted from delayed egg-deposition or embryonic development of *C*. *napi* eggs. Delayed larval development may have contributed to the low larval instar index in S30, L122 Campala and Goe1991. Additionally, numbers of larvae in stems of the tested genotypes / cultivars were positively correlated with the larval instar index. This suggests that ovipositing females of *C*. *napi* are well adapted to select most suitable host plants for their larvae.

### Phenotypic differences between plant genotypes

#### Physical plant traits and their effects on *C*. *napi* larvae

Plant growth stages and the length of plant stems have been found to affect the host plant acceptance of *C*. *napi* [7, 42, 51]. Schaefer-Koesterke et al. [7] and Buechi [42] reported that stems exceeding 20 cm in length were less preferred by *C*. *napi* females for oviposition compared to short stemmed plants. In the present study, there was no evidence that growth stage was associated with low levels of infestation by *C*. *napi*. In samples collected on March 31^st^, the growth of cultivars Express617 and Visby and line S30 was further developed compared to the other genotypes, but Express617 and Visby both contained significantly more larvae than S30. Stem length is not regarded likely to be the plant trait responsible for resistance in S30as stem length of line S30 did not significantly differ from other genotypes on March 31^st^, but contained the least number of *C*. *napi* larvae on May 8^th^ (Fig 1 and Table 4). Additionally, the basal stem diameter, a further physical plant trait, did not appear to have any influence on relative resistance to *C*. *napi* infestation and performance.

#### Glucosinolate profiles

Stems were analysed in order to determine the influence of their glucosinolate content on host plant acceptance by females of *C*. *napi*. As low plant densities did not allow the assessment of the number of deposited eggs in this experiment, the number of larvae per stem was used to estimate resistance to ovipositing females. The dispersion of *C*. *napi* larvae between plant genotypes and cultivars was not closely associated with the content of glucosinolates of non-infested stems (Fig 5). As described in the open-field study previously by Schaefer-Koesterke et al. [7], the number of *C*. *napi* eggs at peak egg abundance was associated with the levels of the glucosinolates glucoalyssin, gluconasturtiin, glucobrassicanapin, glucobrassicin and neoglucobrassicin of non-infested stems. However, in the present study there was no evidence that glucosinolate profiles were associated with the abundance of *C*. *napi* larvae. Wounding of the plant by other insect pests may alter the glucosinolate content of brassicaceous plants [37, 38], therefore insect-proof gauze cages were used to avoid these infestations. In the open-field study plants might have displayed different glucosinolate profiles compared to the present study as a reaction to wounding by other brassicaceous pests, such as *P*. *chrysocephala* or *C*. *pallidactylus*. Hervé et al. [52] reported that the glucosinolate profile in buds of OSR was also not associated with feeding or oviposition in pollen beetle (*Meligethes aeneus* F.). Additionally, there was no evidence that the content of glucosinolates in non-infested stems was linked to larval performance of *C*. *napi* as measured by the larval instar index. Eickermann et al. [39] found that the glucosinolate 4OH in leaves was negatively correlated with larval feeding of *C*. *pallidactylus*, a sympatric species of *C*. *napi*. Ulmer and Dosdall [53] reported that high contents of specific glucosinolates, such as GNA were associated with an increased development time of the cabbage seedpod weevil larvae (*Ceutorhynchus obstrictus* (Mrsh.)). Additionally, Gols et al. [54] suggested that NEO might play a role in reducing the performance of herbivores of brassicaceous plants. However, some *Brassica* specialist herbivores are able to detoxicate glucosinolates [55]. This feature might explain why glucosinolate profiles of non-infested stems were not associated with the performance of *C*. *napi* larvae.

Furthermore, the flavonoid kaempferol has been recognised to reduce infestation by cabbage seed weevil (*Ceutorhynchus obstrictus* (Marsh.)) [56] and crucifer-specific phytoalexins are discussed for their importance for infestation by *D*. *radicum* [57].

This study has shown that different cultivars and lines of OSR have significant effects on infestation and performance of *C*. *napi* larvae. The resynthesized line S30 showed a very low larval infestation and the most delayed larval development rate. The line S30 is a promising candidate as a potential source for breeding cultivars of OSR with resistance to *C*. *napi*. The resistance of S30 seems to be antixenotic and antibiotic, due to very low larval counts and lower larval performance in this line. The glucosinolates were not linked to larval infestation and larval performance. Therefore, other host plant factors as yet underdetermined appear to affect larval infestation and depress larval development in S30. Future work should centre upon the identification of these host plant parameters and should analyse potential effects of further chemical traits, such as flavonoids (e.g. kaempferol) and phytoalexins, or the mechanical strength of the stem pith tissue.

## References

[1] Alford DV, Nilsson C, Ulber B. Insect pests of oilseed rape crops In: Alford DV, editor. Biocontrol of oilseed rape pests. Oxford: Blackwell Science Ltd; 2003 p. 9–42.

[2] Büchi R. Neue Bekämpfungsschwelle für den Rapsstengelrüssler *Ceutorhynchus napi* Gyll. Mitt Schweizerischen Landwirtsch. 1988;35:110–117. German.

[3] Nauen R, Zimmer CT, Andrews M, Slater R, Bass C, Ekbom B, et al Target-site resistance to pyrethroids in European populations of pollen beetle, *Meligethes aeneus* F. Pestic Biochem Physiol. 2012;103: 173–180.

[4] Zimmer CT, Köhler H, Nauen R. Baseline susceptibility and insecticide resistance monitoring in European populations of *Meligethes aeneus* and *Ceutorhynchus assimilis* collected in winter oilseed rape. Entomol Exp Appl. 2014;150: 279–288.

[5] Eickermann M, Beyer M, Görgen K, Hoffman L, Junk J. Shifted migration of the rape stem weevil *Ceutorhynchus napi* (Coleoptera: Curculionidae) linked to climate change. Eur J Entomol. 2014;111: 243–250.

[6] Gao YL, Reitz SR. Emerging themes in the understanding of species displacements. Annu Rev Entomol. 2017,62: 165–183. doi: 10.1146/annurev-ento-031616-035425 27860525 10.1146/annurev-ento-031616-035425

[7] Schaefer-Koesterke HL, Brandes H, Ulber B, Becker HC, Vidal S. The potential of resynthesized lines to provide resistance traits against rape stem weevil in oilseed rape. J Pest Sci. 2017;90: 87–101.

[8] Diederichsen E, Sacristan M. Resynthesis of amphidiploid Brassica species and their clubroot disease reaction. Proceedings of the 8th Rapeseed Congress; 1991 Jul 9–11; Saskatoon, Canada.

[9] Rygulla W, Snowdon R, Eynck C, Koopmann B, von Tiedemann A, Lühs W, et al Broadening the genetic basis of *Verticillium longisporum* resistance in *Brassica napus* by interspecific hybridization. Phytopathology. 2007;97: 1391–1396. doi: 10.1094/PHYTO-97-11-1391 18943507 10.1094/PHYTO-97-11-1391

[10] Eickermann M, Ulber B. Screening of oilseed rape and other brassicaceous genotypes for susceptibility to *Ceutorhynchus pallidactylus* (Mrsh.). J Appl Entomol. 2010;134: 542–550.

[11] Girke A, Schierholt A, Becker HC. Extending the rapeseed genepool with resynthesized *Brassica napus* L. I: Genetic diversity. Genet Resour Crop Evol. 2012;59: 1441–1447.

[12] Olsson G, Ellerstrom S, Tsunoda S, Hinata K, Gomez-Campo C. Polyploidy breeding in Europe In: Tsunoda S, Hinata K, Gomez-Campo C, editors. Brassica crops and wild allies. Tokyo: Scientific Society Press; 1980 p. 167–190.

[13] Cleemput S, Becker HC. Genetic variation in leaf and stem glucosinolates in resynthesized lines of winter rapeseed (*Brassica napus* L.). Genet Resour Crop Evol. 2012;59: 539–546.

[14] Ali JG, Agrawal AA. Specialist versus generalist insect herbivores and plant defense. Trends Plant Sci. 2012;17: 293–302. doi: 10.1016/j.tplants.2012.02.006 22425020 10.1016/j.tplants.2012.02.006

[15] Giamoustaris A, Mithen R. The effect of modifying the glucosinolate content of leaves of oilseed rape (*Brassica napus* ssp. oleifera) on its interaction with specialist and generalist pests. Ann Appl Biol. 1995;126: 347–363.

[16] Kogan M, Ortman EF. Antixenosis—a new term proposed to define Painters nonpreference modality of resistance. Bull Entomol Soc Am. 1978;24: 175–176.

[17] Sarfraz M, Dosdall LM, Keddie B. Diamond-back moth—host plant interactions: implications for pest management. Crop Prot. 2006;25: 625–639.

[18] Barari H, Cook SM, Clark SJ, Williams IH. Effect of a turnip rape (*Brassica rapa*) trap crop on stem-mining pests and their parasitoids in winter oilseed rape (*Brassica napus*). Biol Control. 2005;50: 69–86.

[19] Nilsson C. Trap plants to avoid insecticide application against pollen beetles in oilseed rape. IOBC WPRS Bull. 2004;27(10):215–221.

[20] Büchi R. Mortality of pollen beetle (*Meligethes* spp.) larvae due to predators and parasitoids in rape fields and the effect of conservation strips. Agric Ecosys Environ. 2002;90: 255–263.

[21] Kovács G, Kaasik R, Kaart T, Metspalu L, Luik A, Veromann E. In search of secondary plants to enhance the efficiency of cabbage seed weevil management. Biol Control. 2017;62: 29–38.

[22] Nilsson C. Impact of soil tillage on parasitoids of oilseed rape pests In: Williams IH, editor. Biocontrol-based integrated managment of oilseed rape pests. Dordrecht, Heidelberg, London, New York: Springer Science and Business Media B.V.; 2010 p. 305–312.

[23] Baum JA, Bogaert T, Clinton W, Heck GR, Feldmann P, Ilagan O, et al Control of coleopteran insect pests through RNA interference. Nat Biotechnol. 2007;25: 1322–1326. doi: 10.1038/nbt1359 17982443 10.1038/nbt1359

[24] Ballanger Y. Nuisibilité du charançon de la tige du colza. Phytoma. 1987;384: 35–37. French.

[25] Günthart E. Beiträge zur Lebensweise und Bekämpfung von *Ceutorrhynchus quadridens* Panz. und *Ceutorrhynchus napi* Gyll. mit Beobachtungen an weiteren Kohl- und Rapsschädlingen. Mitt Schweiz Entomol Ges. 1949;22: 441–591. German.

[26] Williams IH. The major insect pests of oilseed rape in Europe and their management: an overview In: Williams IH, editor. Biocontrol-based integrated managment of oilseed rape pests. Dordrecht, Heidelberg, London, New York: Springer Science and Business Media B.V.; 2010 p. 1–43.

[27] Barari H, Cook SM, Williams H. Rearing and identification of the larval parasitoids of *Psylliodes chrysocephala* and *Ceutorhynchus pallidactylus* from field-collected specimens. IOBC WPRS Bull. 2004;27(10):263–272.

[28] Dechert G, Ulber B. Interactions between the stem-mining weevils *Ceutorhynchus napi* Gyll. and *Ceutorhynchus pallidactylus* (Marsh.) (Coleoptera: Curculionidae) in oilseed rape. Agric For Entomol. 2004;6: 193–198.

[29] Ferguson AW, Kenward M, Williams IH, Clark S, Kelm M, Duzic A. Interactions between the cabbage seed weevil (Ceutorhynchus assimilis Payk.) and the brassica pod midge (Dasineura brassicae Winn.) infesting oilseed rape pods. Proceedings of the 9th International Rapeseed Congress; 1995 Jul 4–7; Cambridge, United Kingdom.

[30] Riggin-Bucci TM, Gould F. Effects of surfactants, *Bacillus thuringiensis* formulations and plant damage on oviposition by diamond-back moth (Lepidoptera: Plutellidae). J Econ Entomol. 1996;89: 891–897.

[31] Rojas JC. Influence of host plant damage on the host finding behavior of *Mamestra brassicae* (Lepidoptera: Noctuidae). Environ Entomol. 1999;28: 588–593.

[32] Schütz S, Weissbecker B, Klein B, Hummel HE. Host plant selection of the colorado beetle as influenced by damage induced volatiles of the potato plant, Naturwissenschaften. 1997;84: 212–217.

[33] Cook SM, Khan ZR, Picket JA. The use of push-pull strategies in integrated pest managment. Annu Rev Entomol. 2007;52: 375–400. doi: 10.1146/annurev.ento.52.110405.091407 16968206 10.1146/annurev.ento.52.110405.091407

[34] Moyes CL, Raybould AF. The role of spatial scale and intraspecific variation in secondary chemistry in host-plant location by *Ceutorhynchus assimilis* (Coleoptera: Curculionidae). Proc R Soc Lond B Biol Sci. 2001;268: 1567–1573. 10.1098/rspb.2001.1685 PMC1088779 11487403

[35] Renwick JAA. The chemical world of crucivores: lures, treats and traps. Entomol Exp Appl. 2002;104: 35–42.

[36] Hopkins RJ, van Dam NM, van Loon JJA. Role of glucosinolates in insect-plant relationships and multitrophic interactions. Annu Rev Entomol. 2009;54: 57–83. doi: 10.1146/annurev.ento.54.110807.090623 18811249 10.1146/annurev.ento.54.110807.090623

[37] Bartlet E, Williams I, Pickett J, Ellis P, Derridj S. The ideal glucosinolate profile for pest resistance in oilseed rape. IOBC WPRS Bull. 1999;22(10):13–17.

[38] Bodnaryk RP. Effects of wounding on glucosinolates in the cotyledons of oilseed rape and mustard. Phytochemistry. 1992;31: 2671–2677.

[39] Eickermann M, Ulber B, Vidal S. Resynthesized lines and cultivars of *Brassica napus* L. provide sources of resistance to the cabbage stem weevil (*Ceutorhynchus pallidactylus* (Mrsh.)). Bull Entomol Res. 2011;101: 287–294. doi: 10.1017/S0007485310000489 21092380 10.1017/S0007485310000489

[40] Tansey JA, Dosdall LM. (2011) Differential responses by some insect pests to novel insect-resistant Brassica napus L. [CD-ROM]. Proceedings of the 13th International Rapeseed Congress; 2011 Jul 5–9; Prague, Czech Republic.

[41] Siemens DH, Mitchelolds T. Glucosinolates and herbivory by specialists (Coleoptera: Chrysomelidae, Lepidoptera: Plutellidae): consequences of concentration and induced resistance. Environ Entomol. 1996;25: 1344–1353.

[42] Büchi R. Eiablage des Rapsstengelrüsslers *Ceutorhynchus napi* Gyll., in Abhängigkeit der Stengellänge bei verschiedenen Rapssorten. Anz Schädlingskunde Pflanzenschutz Umweltschutz. 1996;69: 136–139. German.

[43] Nuss H. Einfluss der Pflanzendichte und -architektur auf Abundanz und innnerpflanzliche Verteilung stängelminierender Schadinsekten in Winterraps. PhD Thesis, Georg-August Universität. 2004. Available from: https://ediss.uni-goettingen.de/handle/11858/00-1735-0000-0006-AB2E-7?show=full

[44] Girke A. Neue Genpools aus resynthetisiertem Raps (Brassica napus L.) für die Hybridzüchtung. PhD Thesis, Georg-August Universität. 2002. Available from: https://ediss.uni-goettingen.de/handle/11858/00-1735-0000-0006-AEB2-3?locale-attribute=en

[45] Kurtz B, Karlovsky P, Vidal S. Interaction between western corn rootworm (Coleoptera: Chrysomelidae) larvae and root-infecting *Fusarium verticillioides*. Environ Entomol. 2010;39: 1532–1538. doi: 10.1603/EN10025 22546449 10.1603/EN10025

[46] Ferguson AW, Klukowski Z, Walczak B, Clark SJ, Mugglestone MA, Perry JN, et al Spatial distribution of pest insects in oilseed rape: implications for integrated pest management. Agric Ecosys Environ. 2003;95: 509–521.

[47] Lancashire PD, Bleiholder H, Boom T, Langelüddeke P, Stauss R, Weber E, et al A uniform decimal code for growth stages of crops and weeds. Ann Appl Biol. 1991;119: 561–601.

[48] Thies W. Analysis of glucosinolates in seeds of rapeseed (*Brassica napus* L.): Concentration of glucosinolates by ion exchanges. Z Pflanzenzucht. 1977;79: 331–335.

[49] Geladi P, Kowalski B. Partial least squares regression: a tutorial. Anal Chim Acta. 1986;185: 1–17.

[50] Barker E, Rayens W. Partial least squares for discrimination. J Chemom. 2003;17: 166–173.

[51] Lerin J. Influence of the growth rate of oilseed rape on the splitting of the stem after an attack of *Ceutorhynchus napi* Gyll. IOBC WPRS Bull. 1993;16(9):160–163.

[52] Hervé MR, Delourme R, Leclair M, Marnet N, Cortesero AM. How oilseed rape (*Brassica napus*) genotype influences pollen beetle (*Meligethes aeneus*) oviposition. Arthropod Plant Interac. 2014;8: 383–392.

[53] Ulmer BJ, Dosdall LM. Glucosinolate profile and oviposition behavior in relation to the susceptibilities of Brassicaceae to the cabbage seedpod weevil. Entomol Exp Appl. 2006;121: 203–213.

[54] Gols R, Bukovinszky T, van Dam NM, Dicke M, Bullock JM, Harvey JA. Performance of generalist and specialist herbivores and their endoparasitoids differs on cultivated and wild *Brassica* populations. J Chem Ecol. 2008;34: 132–143. doi: 10.1007/s10886-008-9429-z 18231835 10.1007/s10886-008-9429-z PMC2239250

[55] Després L, David J-P, Gallet C. The evolutionary ecology of insect resistance to plant chemicals. Trends Ecol Evol. 2007;22: 298–307. doi: 10.1016/j.tree.2007.02.010 17324485 10.1016/j.tree.2007.02.010

[56] Lee RWH, Malchev IT, Rajcan I, Kott LS. Identification of putative quantitative trait loci associated with a flavonoid related to resistance to cabbage seedpod weevil (*Ceutorhynchus obstrictus*) in canola derived from an intergeneric cross, *Sinapis alba* x *Brassica napus*. Theor Appl Genet. 2014;127: 419–428. 10.1007/s00122-013-2228-0 24231920

[57] Baur R, Städler E, Monde K, Takasugi M. Phytoalexins from Brassica (Cruciferae) as oviposition stimulants for the cabbage root fly, *Delia radicum*. Chemoecology. 1998;8: 163–168.

